# Supplementary material for: Using Gaze for Behavioural Biometrics
Source: Sensors (Basel). 2023 Jan 22;23(3):1262. doi: 10.3390/s23031262 (PMC9920149; doi:10.3390/s23031262)
Supplement: Supplementary file 1 [file sensors-23-01262-s001.zip › sensors-2071809-supplementary.pdf]

Supplementary Materials
Using gaze for behavioural biometrics

Alessandro D'Amelio[1], Sabrina Patania[1], Sathya Bursic[1,2], Vittorio Cuculo[1], and Giuseppe Boccignone[1]

[1] *PHuSe Lab, Department of Computer Science, University of Milano Statale, via Celoria 18,- 20133, Milan, Italy; {alessandro.damelio, sabrina.patania, vittorio.cuculo, giuseppe.boccignone}@unimi.it*
[2] *Department of Psychology, University of Milano-Bicocca, Piazza dell'Ateneo Nuovo, 1 - 20126, Milan, Italy; sathya.bursic@unimib.it*

1 Further analysis of the feature vector components

In this supplementary section we expand on the analysis of the components (features) of vector $\mathbf{v}_{(id),k}$, which characterises the visual behavior of observer id while scrutinising the stimulus k (image), captured as a sequence of events (the fixations and saccades composing the scan path). By viewing the K images in the dataset, each subject id gives rise to a sample $\{\mathbf{v}_{(id),k}\}_{k=1}^{K}$, which is best described by its empirical distribution and summary statistics; at a finer level of analysis, for each component n of the vector, we can consider the distribution and summary statistics of sample $\{\mathbf{v}[n]_{(id),k}\}_{k=1}^{K}$.

The latter are reported in the suitable form of violin plots. Each plot depicts at a glance summary statistics and density (smoothed by a kernel density estimator) of one vector component/feature. In the following Figure S1, panels display the sequence of violin plots of the same component, each plot corresponding to one subject; the feature considered is specified at the top of the panel.

For a quick reference to the meaning of parameters, see Table 2 in the manuscript.

Overall, the figure gives an insight into the nuanced variations of features among subjects.

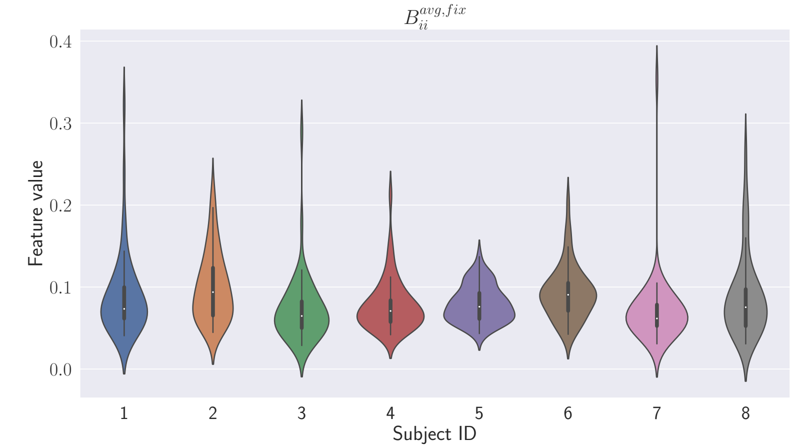
$B_{ii}^{avg,fix}$
0.4
0.3
0.2
0.1
0.0
Feature value
1
2
3
4
5
6
7
8
Subject ID

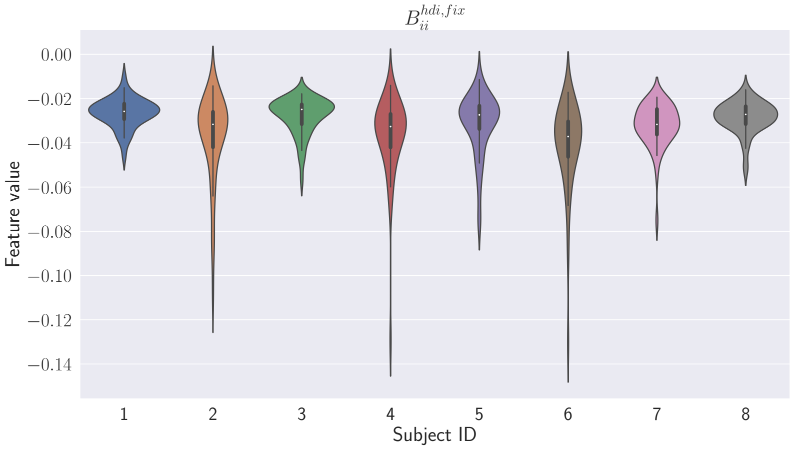
$B_{ii}^{hdi,fix}$
0.00
−0.02
−0.04
−0.06
−0.08
−0.10
−0.12
−0.14
Feature value
1
2
3
4
5
6
7
8
Subject ID

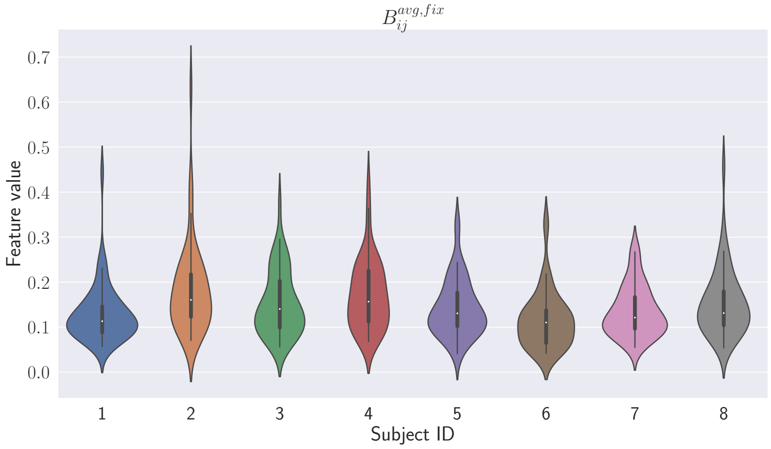
$B_{ij}^{avg,fix}$
0.7
0.6
0.5
0.4
0.3
0.2
0.1
0.0
Feature value
1
2
3
4
5
6
7
8
Subject ID

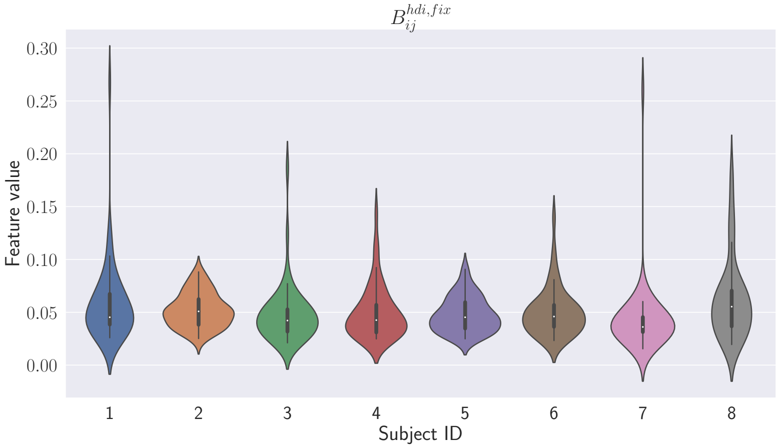
$B_{ij}^{hdi,fix}$
Feature value
0.30
0.25
0.20
0.15
0.10
0.05
0.00
1 2 3 4 5 6 7 8
Subject ID

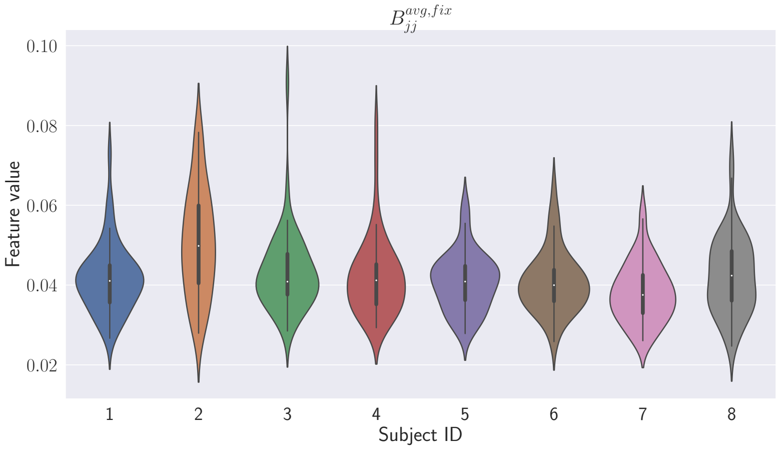
$B_{jj}^{avg,fix}$
Feature value
0.10
0.08
0.06
0.04
0.02
1 2 3 4 5 6 7 8
Subject ID

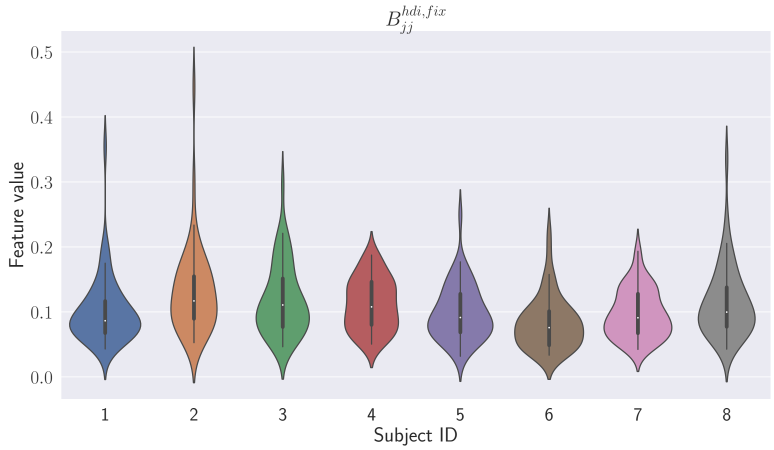
$B_{jj}^{hdi,fix}$
Feature value
0.5
0.4
0.3
0.2
0.1
0.0
1 2 3 4 5 6 7 8
Subject ID

$\Gamma_{ii}^{avg,fix}$

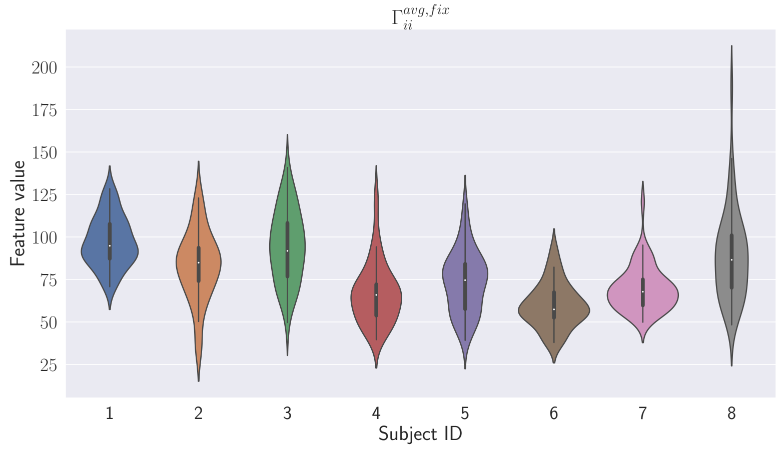

$\Gamma_{ii}^{hdi,fix}$

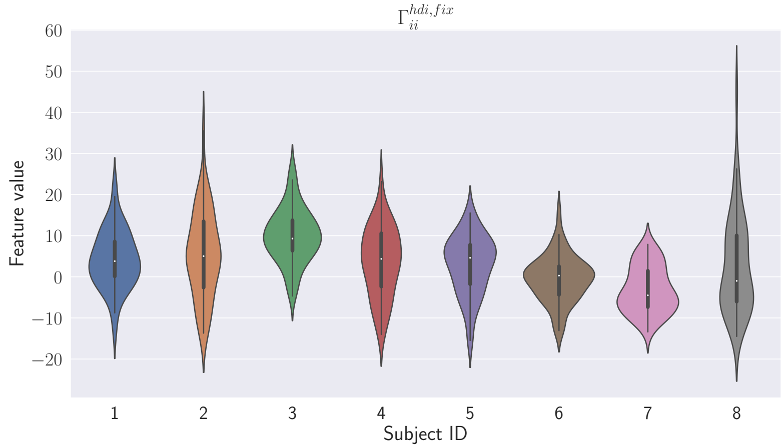

$\Gamma_{ij}^{avg,fix}$

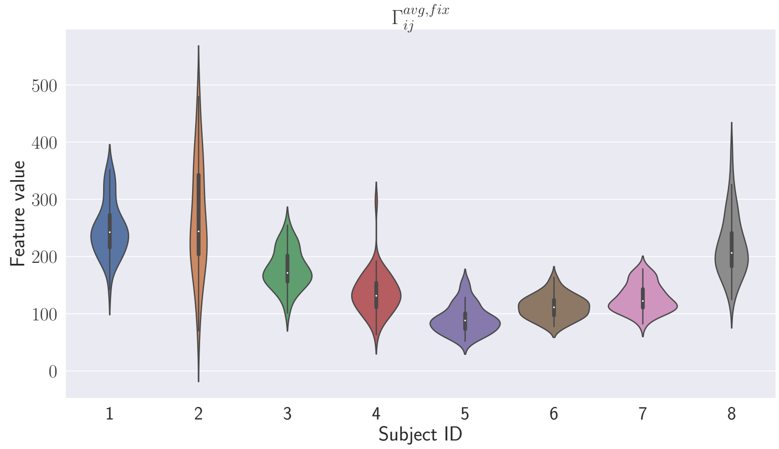

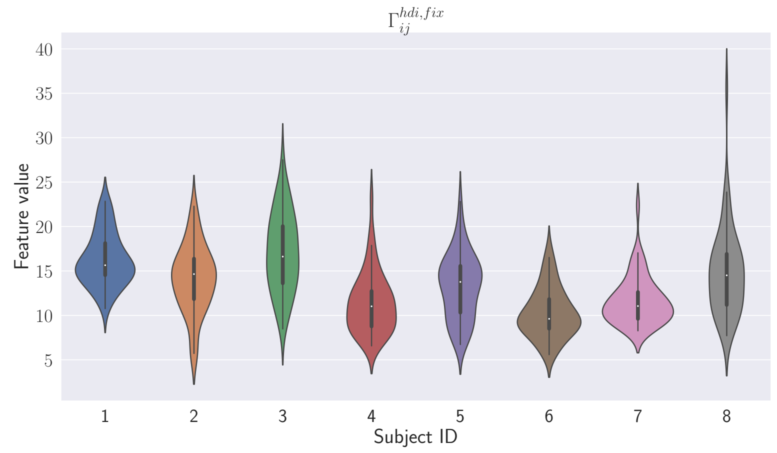
$\Gamma_{ij}^{hdi,fix}$
Feature value
40
35
30
25
20
15
10
5
1
2
3
4
5
6
7
8
Subject ID

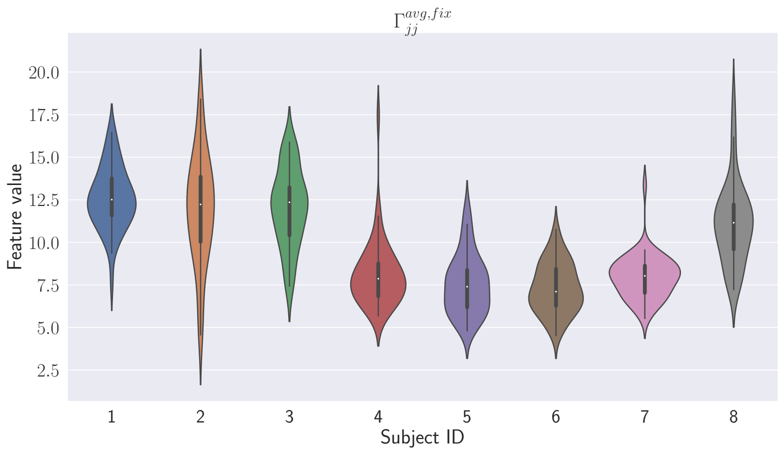
$\Gamma_{jj}^{avg,fix}$
Feature value
20.0
17.5
15.0
12.5
10.0
7.5
5.0
2.5
1
2
3
4
5
6
7
8
Subject ID

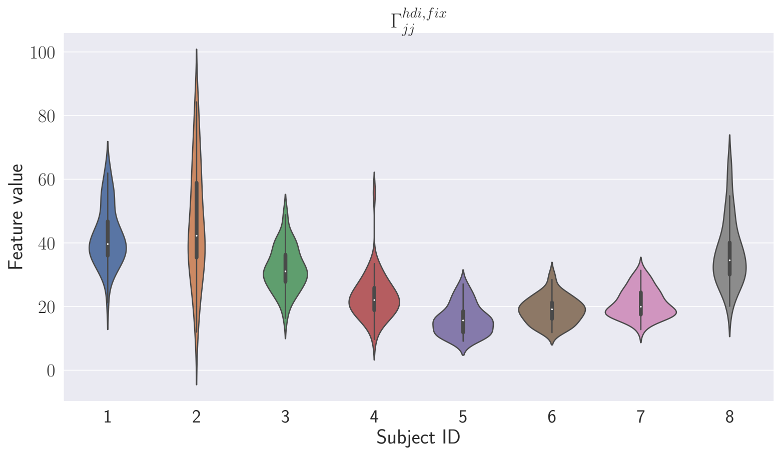
$\Gamma_{jj}^{hdi,fix}$
Feature value
100
80
60
40
20
0
1
2
3
4
5
6
7
8
Subject ID

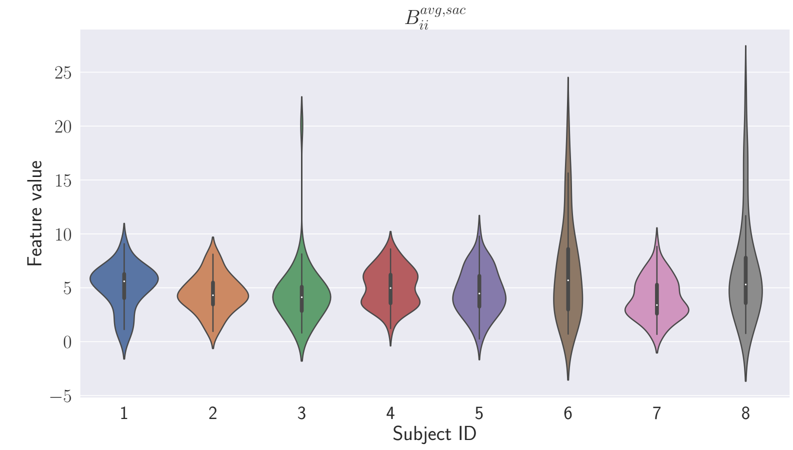
$B_{ii}^{avg,sac}$
Feature value
25
20
15
10
5
0
−5
1
2
3
4
5
6
7
8
Subject ID

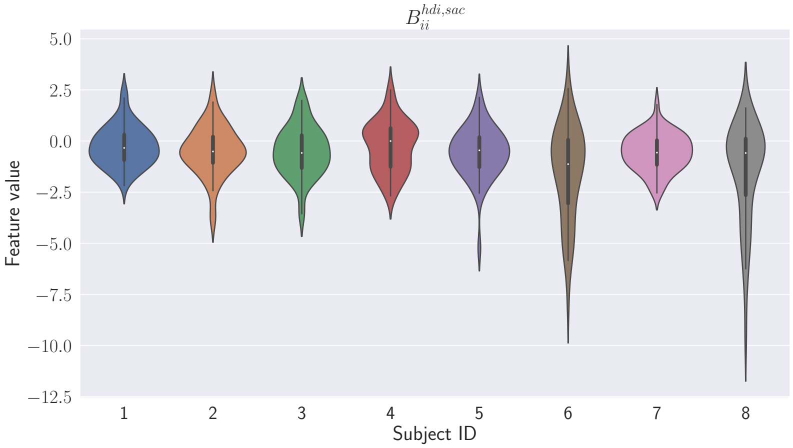
$B_{ii}^{hdi,sac}$
Feature value
5.0
2.5
0.0
−2.5
−5.0
−7.5
−10.0
−12.5
1
2
3
4
5
6
7
8
Subject ID

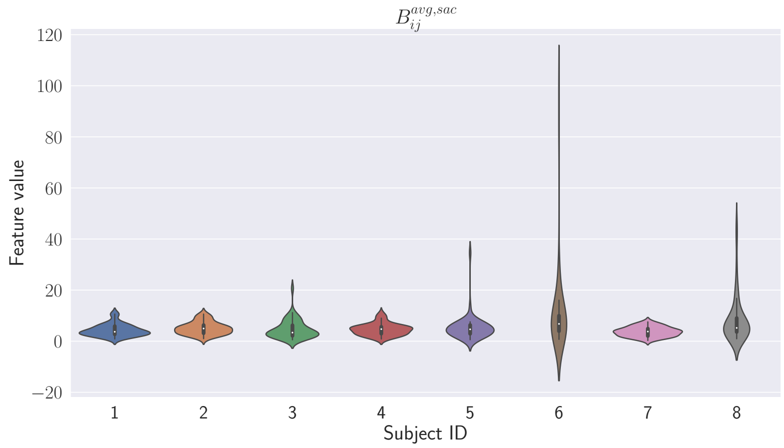
$B_{ij}^{avg,sac}$
Feature value
120
100
80
60
40
20
0
−20
1
2
3
4
5
6
7
8
Subject ID

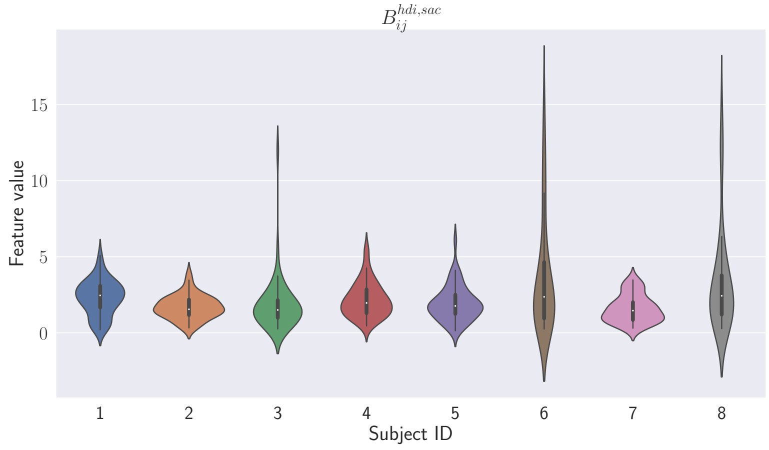
$B_{ij}^{hdi,sac}$
Feature value
Subject ID
1
2
3
4
5
6
7
8
0
5
10
15

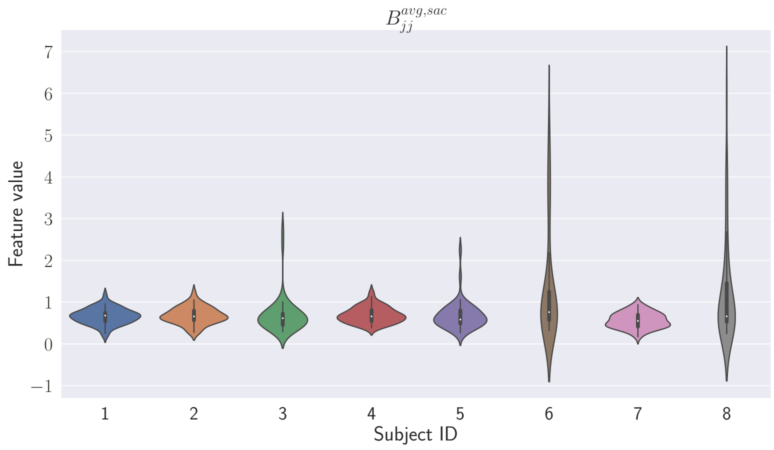
$B_{jj}^{avg,sac}$
Feature value
Subject ID
1
2
3
4
5
6
7
8
−1
0
1
2
3
4
5
6
7

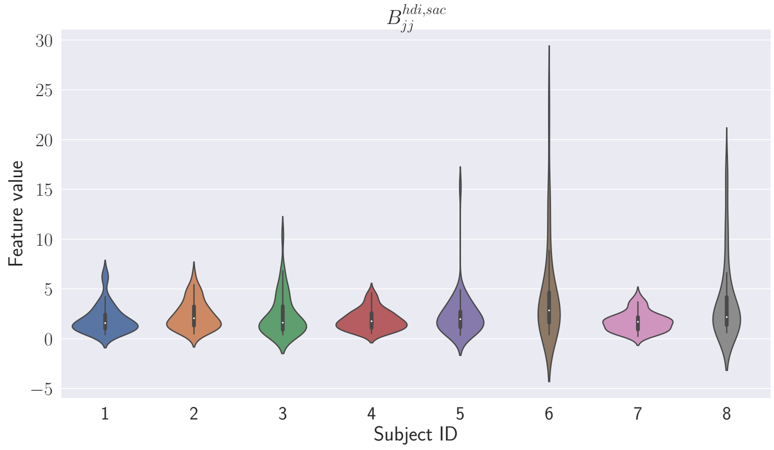
$B_{jj}^{hdi,sac}$
Feature value
Subject ID
1
2
3
4
5
6
7
8
−5
0
5
10
15
20
25
30

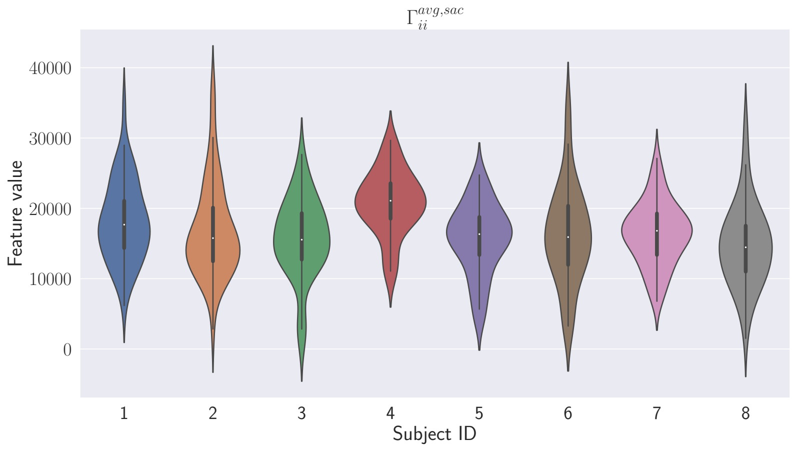
$\Gamma_{ii}^{avg,sac}$
Feature value
40000
30000
20000
10000
0
1
2
3
4
5
6
7
8
Subject ID

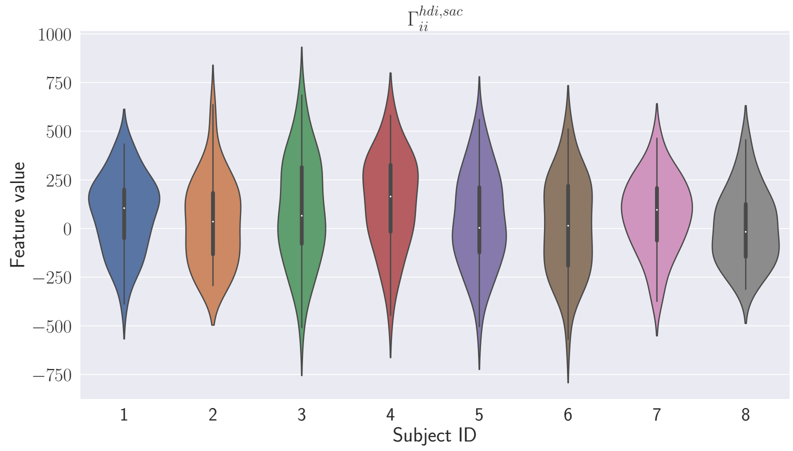
$\Gamma_{ii}^{hdi,sac}$
Feature value
1000
750
500
250
0
−250
−500
−750
1
2
3
4
5
6
7
8
Subject ID

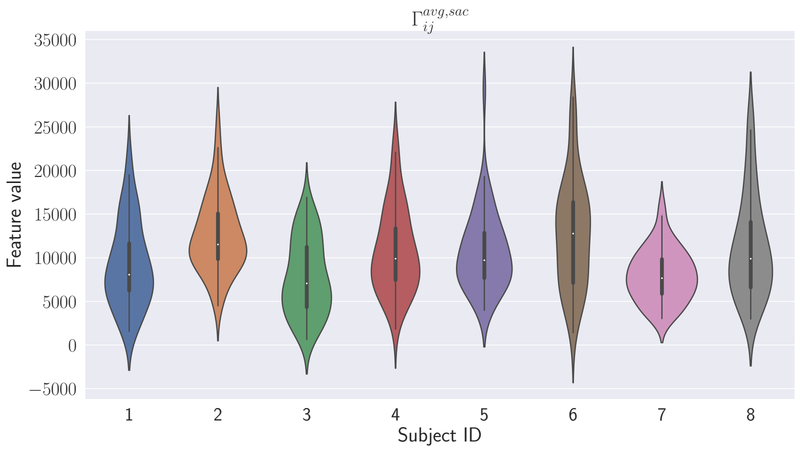
$\Gamma_{ij}^{avg,sac}$
Feature value
35000
30000
25000
20000
15000
10000
5000
0
−5000
1
2
3
4
5
6
7
8
Subject ID

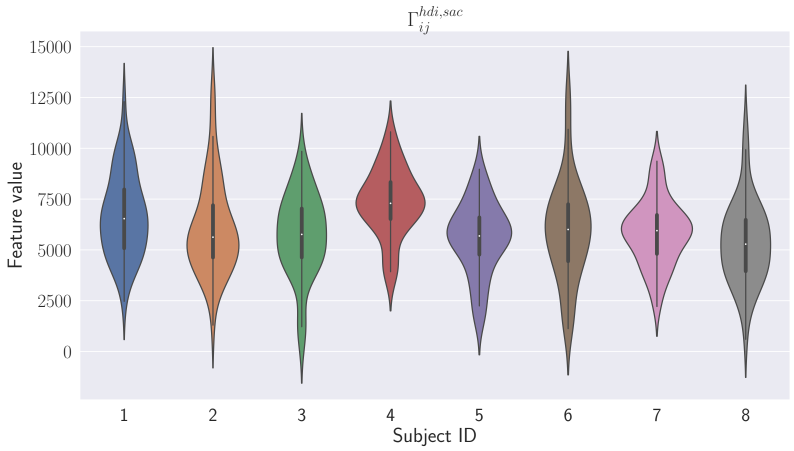
$\Gamma_{ij}^{hdi,sac}$
Feature value
15000
12500
10000
7500
5000
2500
0
1
2
3
4
5
6
7
8
Subject ID

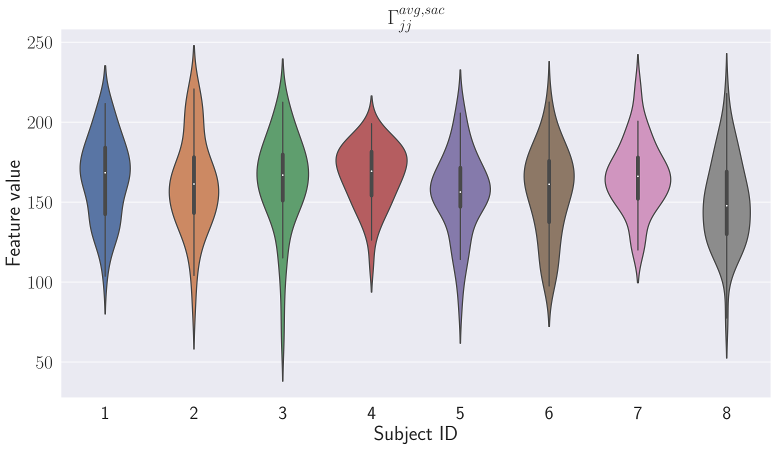
$\Gamma_{jj}^{avg,sac}$
Feature value
250
200
150
100
50
1
2
3
4
5
6
7
8
Subject ID

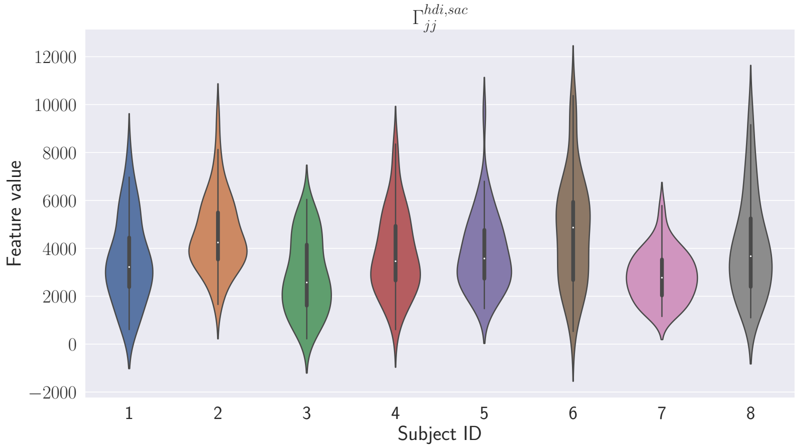

Figure S1: Violin plots for the distributions of the values assumed by each of the 24 considered features for each subject.
